# Book-embedded hair reveals mineral-rich diets among urban commoners in early modern to modern Japan

**DOI:** 10.1038/s41598-026-63908-y

**Published:** 2026-08-17

**Authors:** Atsushi Maruyama, Shota Kuwaki, Shuntaro Kimura, Yuki Sanada, Masakazu Oikawa, Shoji Futatsukawa, Yukihiro Kohmatsu, Atsushi Iriguchi

**Affiliations:** 1 Faculty of Advanced Science and Technology, Ryukoku University, Seta-oe, Otsu, 520-2194 Shiga Japan; 2 National Institute for Quantum Science and Technology, Institute for Quantum Medical Science, 4-9-1 Anagawa, Inage-ku, Chiba, 263-8555 Japan; 3 Alpha Tau Medical K.K., 1-9-15 Nihonbashimuromachi, Chiyoda-ku, Tokyo, 103-0022 Japan; 4 The Ministry of the Environment, Kyushu Regional Environment Office, 4F, Kumamoto Regional Joint Government Building B, Kasuga 2-10-1, Nishi Ward, Kumamoto City, 860-0047 Kumamoto Prefecture Japan; 5 National Institute of Japanese Literature, Midori-cho, Tachikawa, Tokyo, 190-0014 Japan

**Keywords:** Ecology, Ecology, Environmental sciences, Evolution, Ocean sciences

## Abstract

Urban populations in early modern to modern Japan depended strongly on rice and marine fish, raising the question of whether such apparently simple diets provided sufficient mineral availability in everyday life. Here, we analyze human hair preserved in historical book covers to reconstruct elemental exposure among urban commoners from the seventeenth to nineteenth centuries. Screening approximately 70,000 volumes allowed individual hair samples to be linked to specific cities and years through colophon records, enabling a rare population-scale reconstruction from abundant and well-dated materials. Multi-element analyses showed that several elements, including essential ones, were consistently higher in historical hair than in contemporary reference populations, indicating greater mineral availability in past diets. Carbon and nitrogen isotope data confirmed strong reliance on C_3_ staples, particularly rice, with marine fish providing a major and increasing nitrogen source. Interpreted together, the elemental and isotopic patterns suggest effects of rice polishing, marine resources, salt preservation, cookware, and other material practices. These findings show that historical books can preserve bioarchives for reconstructing everyday diet and material culture, and they indicate that urban commoners in early modern to modern Japan had substantially greater dietary mineral availability than contemporary populations, despite apparently simple diets centered on rice and marine fish.

**Supplementary Information:**

The online version contains supplementary material available at 10.1038/s41598-026-63908-y.

## Introduction

Mineral availability has been a persistent determinant of human nutrition, yet its long-term historical dynamics remain poorly understood. Mineral deficiencies still affect more than two billion people worldwide^1,2^, and in Japan the intake of several essential minerals remains below recommended levels in contemporary populations^3,4^. Most discussions of this problem focus on contemporary food systems or on evolutionary contrasts with hunter-gatherer diets^5–7^, but much less is known about how mineral availability changed within later agricultural societies. This gap limits our ability to place present-day micronutrient insufficiency in a longer historical context and to identify the cultural and technological processes that have shaped mineral availability in everyday diets.

Reconstructing historical mineral nutrition is challenging because few archives preserve both biological material and reliable temporal context. Bones and teeth provide valuable long-term records, but they integrate information over broad portions of the life course and are often constrained by sample availability, preservation, and ethical considerations^8,9^. Hair offers a complementary archive because it records dietary and environmental signals over shorter timescales and is well suited to both multi-element and stable isotope analyses^10–14^. However, well-dated historical hair samples are rare, and this limitation has hindered population-scale reconstructions of past dietary mineral availability.

**Fig. 1:**
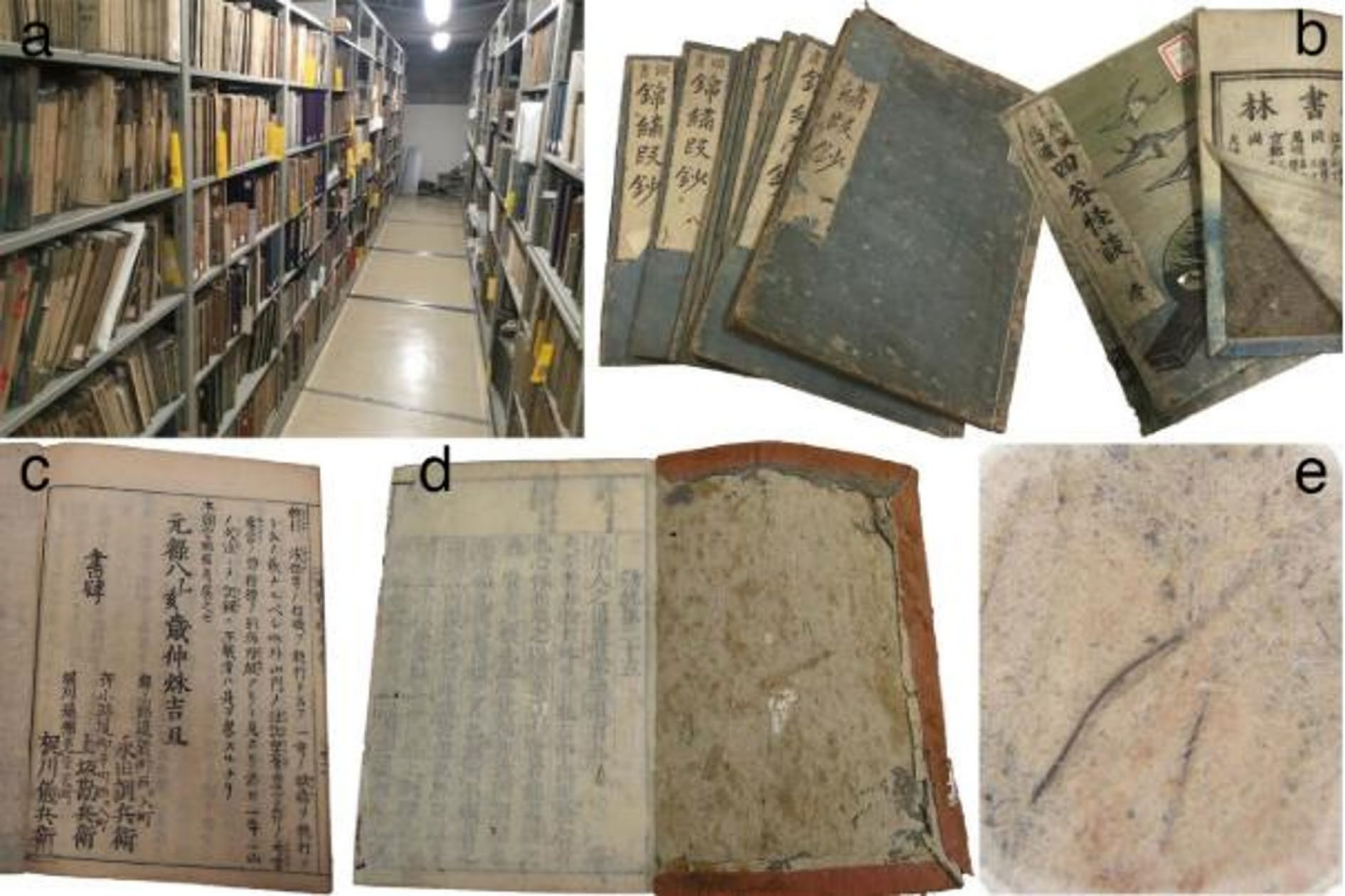
Historical books preserving embedded hair. a: Shelves at Ryukoku University Omiya Library that house approximately 70,000 volumes printed in early modern to modern Japan. b: Two sets of traditional Japanese books. c: The colophon, usually found on the last page, which provides bibliographic information such as the year and place of publication, but does not necessarily indicate the actual year and place of printing. d: Recycled thick paperboard used exclusively for the front and back covers, in which hair is embedded; this board is originally covered with high-quality paper. e: Close-up of hair embedded in the recycled thick paperboard.

In early modern to modern Japan (17th to 19th centuries), human hair embedded in the recycled paperboard of historical book covers provides an unusual archive for reconstructing past diets (Fig. 1). Because such books often preserve colophon information on year and place of publication, the associated hair can be anchored to specific historical contexts at a scale rarely achievable with other biological materials. Our previous pilot study using stable isotope analysis of book-embedded hair showed that urban diets in this period relied strongly on C_3_ staples, especially rice, together with marine fish as a dominant nitrogen source^15^. This pattern contrasts with isotope data from contemporary Japanese populations^16,17^, which reflect dietary inputs from more diversified food sources^16–20^, while remaining broadly consistent with historical scholarship emphasizing the centrality of rice and the relatively limited role of mammalian meat in Japanese food culture^21,22^. This finding raises the further question of whether such apparently simple diets were nonetheless sufficient to sustain substantial mineral availability.

Here, we use particle-induced X-ray emission (PIXE)-based multi-element analysis of hair identified through screening of approximately 70,000 volumes, complemented by stable isotope measurements that provide dietary context, to reconstruct elemental patterns in early modern to modern Japan. Because the hairs were embedded in recycled paperboard and traditional papermaking materials were not mineral-rich, contamination is unlikely to have been a major influence. Contrary to the expectation that such apparently simple diets might have been mineral-poor, we find that historical hair contains substantially higher concentrations of multiple elements, including essential ones, than contemporary reference populations, indicating markedly different elemental exposure associated with everyday diets. These elemental patterns, interpreted together with isotope evidence, point to the combined importance of rice polishing, marine resources, salt preservation, cookware, and other material practices in shaping mineral availability. More broadly, our results show that historical books can preserve well-dated bioarchives for reconstructing long-term interactions among diet, environment, and culture.

## Results

### Historical hair reveals mineral-rich diets relative to today

**Fig. 2:**
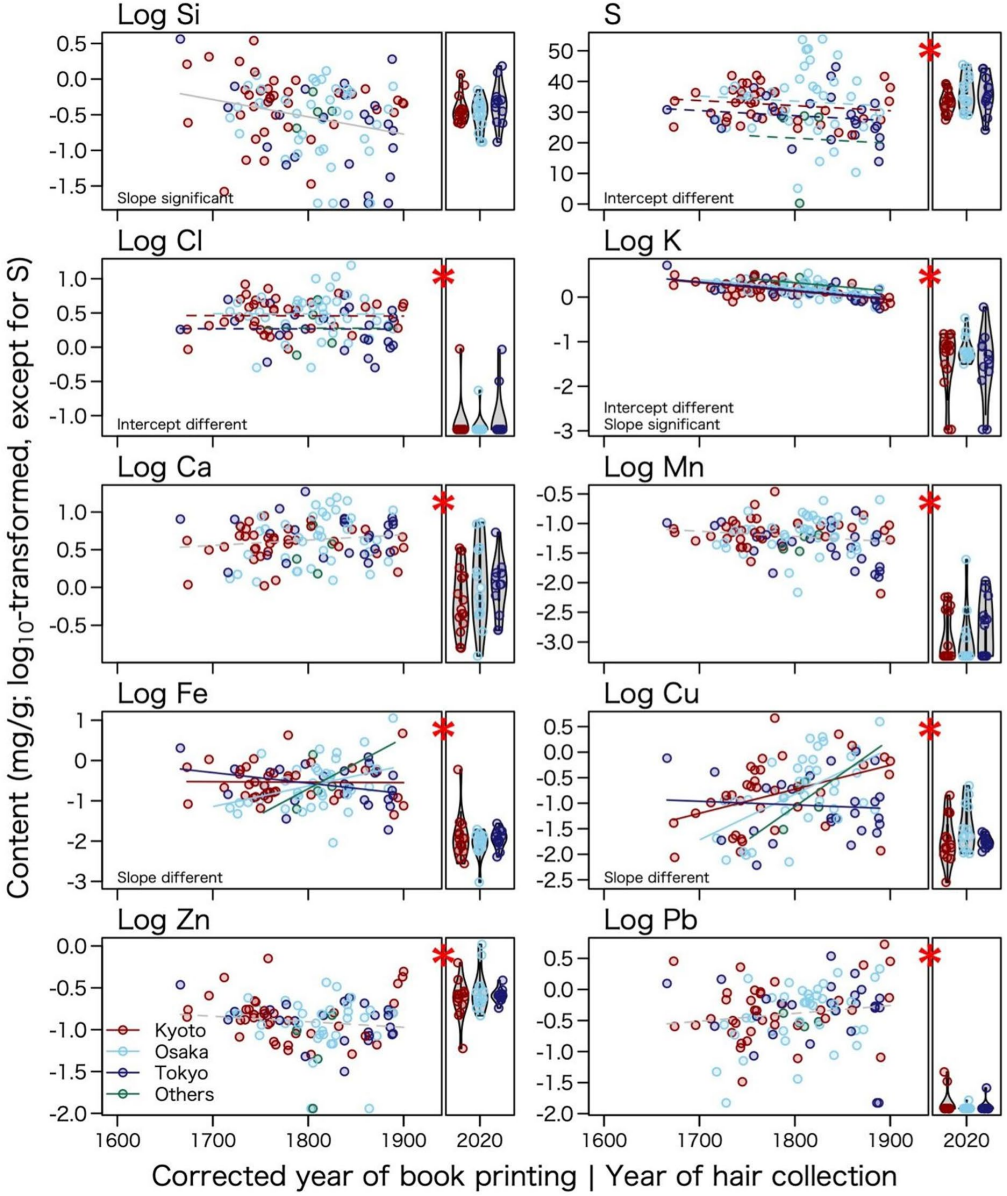
Elemental contents (log_10_-transformed, except for S) in human hair measured by PIXE analysis, plotted against the bibliographically corrected year of woodblock printing or hair collection. Scatter plots (left) represent hair embedded in book paper, and violin plots (right) represent contemporary hair (2020). Colors indicate the different locations (Kyoto, Osaka, Tokyo, and five other provinces). Lines represent the results of linear models. Multiple lines are shown only when intercepts or slopes differ significantly between locations. Solid lines are used when slopes are significantly different from zero or different between locations. Red asterisks between the scatter and violin plots indicate significant differences between the two eras (hair embedded in book paper and contemporary hair).

Elemental concentrations in human hair preserved in historical book covers were quantified by PIXE in 104 book sets for ten elements: silicon (Si), sulfur (S), chlorine (Cl), potassium (K), calcium (Ca), manganese (Mn), iron (Fe), copper (Cu), zinc (Zn), and lead (Pb). Linear models comparing 104 historical and 40 contemporary samples from major Japanese cities revealed consistent differences for nine elements, with no difference detected for Si (Fig. 2). Historical hair showed significantly higher concentrations of Cl (*F*_1,134_ = 814.79, *p* < 0.001), K (*F*_1,136_ = 505.39, *p* < 0.001), Ca (*F*_1,136_ = 121.39, *p* < 0.001), Mn (*F*_1,134_ = 647.81, *p* < 0.001), Fe (*F*_1,136_ = 232.81, *p* < 0.001), Cu (*F*_1,136_ = 48.73, *p* < 0.001), and Pb (*F*_1,136_ = 346.53, *p* < 0.001) than contemporary hair, whereas S (*F*_1,136_ = 4.73, *p* = 0.031) and Zn (*F*_1,136_ = 38.53, *p* < 0.001) were higher in contemporary hair (Fig. 2). These results indicate that urban populations in early modern to modern Japan were exposed to higher levels of multiple elements derived from dietary and environmental sources than those observed in contemporary reference populations.

The same linear models detected no significant main effects of location for any element (*p* > 0.05). Interaction effects between era and location were significant for Mn (*F*_2,134_ = 4.147, *p* = 0.018) and Cl (*F*_2,134_ = 3.280, *p* = 0.041), indicating that the magnitude of historical–contemporary differences varied modestly among cities.

### Temporal trajectories and spatial differences in elemental concentrations

Linear models applied to the historical dataset showed that several elements exhibited significant temporal trends (Fig. 2). K showed a strong decline over time (*t* = − 7.16, *p* < 0.001), and Si also decreased modestly (*t* = − 2.09, *p* = 0.039). These results indicate gradual shifts in the elemental composition of hair across the study period. A multivariate summary of the historical samples based on Aitchison distances calculated from centered log-ratio–transformed elemental profiles also indicates a temporal shift in overall elemental composition (Fig. S1), but the loadings of individual elements are distributed across multiple directions in ordination space, indicating limited dimensional reduction and supporting the element-specific analyses presented in Fig. 2.

Spatial differences among locations were generally limited. Relative to Kyoto, S concentrations were lower in the five other provinces (*t* = − 2.28, *p* = 0.025), Cl concentrations were lower in Tokyo (*t* = − 2.44, *p* = 0.017), and K concentrations were higher in the five other provinces (*t* = 2.47, *p* = 0.015). For most elements, however, no significant location effects were detected.

A few elements exhibited significant interactions between year and location. Fe decreased over time in Kyoto but increased in Osaka (*t* = 2.27, *p* = 0.026), whereas Cu increased in Kyoto but decreased in Tokyo (*t* = − 2.10, *p* = 0.038). These patterns suggest that while overall temporal trends were broadly shared, certain elements followed city-specific trajectories.

### Isotopic evidence of diet and its links to elemental concentrations

**Fig. 3:**
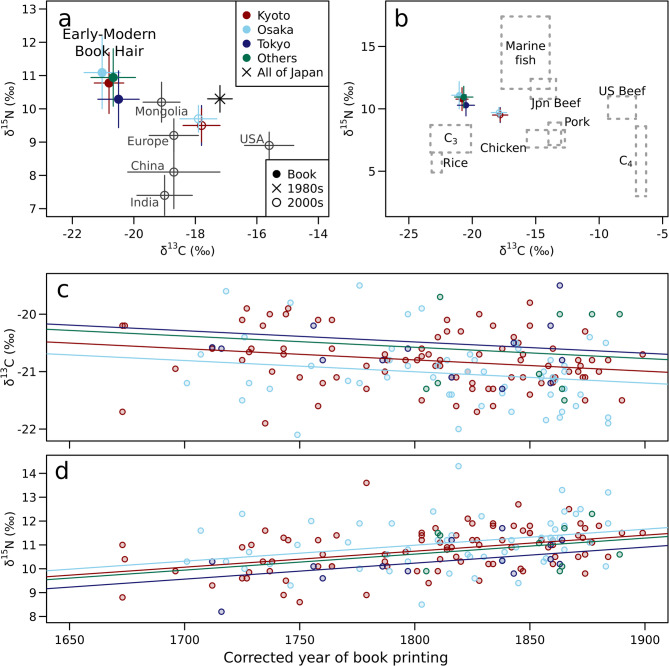
Carbon and nitrogen stable isotope ratios (*δ*^13^C and *δ*^15^N) of hair embedded in book paper (solid circles). a, b: *δ*^13^C–*δ*^15^N plots (means ± SD), shown with hair of contemporary (2000s) people (open circles and cross)^16–20^(a) and with contemporary (2000s) foods (dashed-line rectangles represent means ± SD area)^16,17,26,27^(b). c, d: Temporal changes in *δ*^13^C (c) and *δ*^15^N (d), plotted against the bibliographically corrected year of woodblock printing. Contemporary isotope ratios were adjusted to the atmospheric baseline of the 17th to 19th centuries to account for the Suess effect^23–25^. Trophic discrimination factors specific to human hair (*Δ*^13^C = 2.5‰, *Δ*^15^*N* = 4.15‰)^16^ are added to the foods’ values. Colors represent different locations of book printing. Lines indicate regressions.

Stable isotope ratios of carbon and nitrogen in historical hair from 161 book sets provide context for interpreting the observed elemental patterns. After correction for the Suess effect^23–25^, hair from the early modern to modern period exhibited lower *δ*^13^C and higher *δ*^15^N values than contemporary Japanese hair (*Δ*^13^C = 3.0‰, *t* = − 68.38, df = 160, *p* < 0.001; *Δ*^15^*N* = 1.4‰, *t* = 18.62, df = 160, *p* < 0.001; Fig. 3a)^16,17^. For broader reference, these *δ*^13^C and *δ*^15^N were also lower and higher, respectively, than contemporary populations from Asia, Europe, and the USA^17–20^. These values are consistent with diets dominated by C_3_ plant staples, particularly rice, together with substantial inputs of marine fish nitrogen (Fig. 3b)^16,17,26,27^.

Linear models applied to historical isotope data further suggest gradual shifts in dietary composition within the historical period. Hair *δ*^13^C declined significantly through time across locations (*t* = − 2.43, *p* = 0.016; Fig. 3c), indicating a progressive reduction in C_4_ plant inputs such as millet and foxtail millet. Differences among cities were modest: *δ*^13^C intercepts were lower in Osaka (*t* = − 2.17, *p* = 0.031) and higher in Tokyo (*t* = 2.01, *p* = 0.046) relative to Kyoto, whereas the five other provinces did not differ significantly (*t* = 1.15, *p* > 0.10). In contrast, *δ*^15^N increased significantly over time (*t* = 4.97, *p* < 0.001; Fig. 3d), consistent with increasing reliance on ^15^N-enriched nitrogen sources, mainly marine fish. Slopes did not differ among locations, and only a marginal difference in intercepts was detected between Kyoto and Tokyo (*t* = − 1.92, *p* = 0.056), implying slightly lower *δ*^15^N values in Kyoto.

**Fig. 4:**
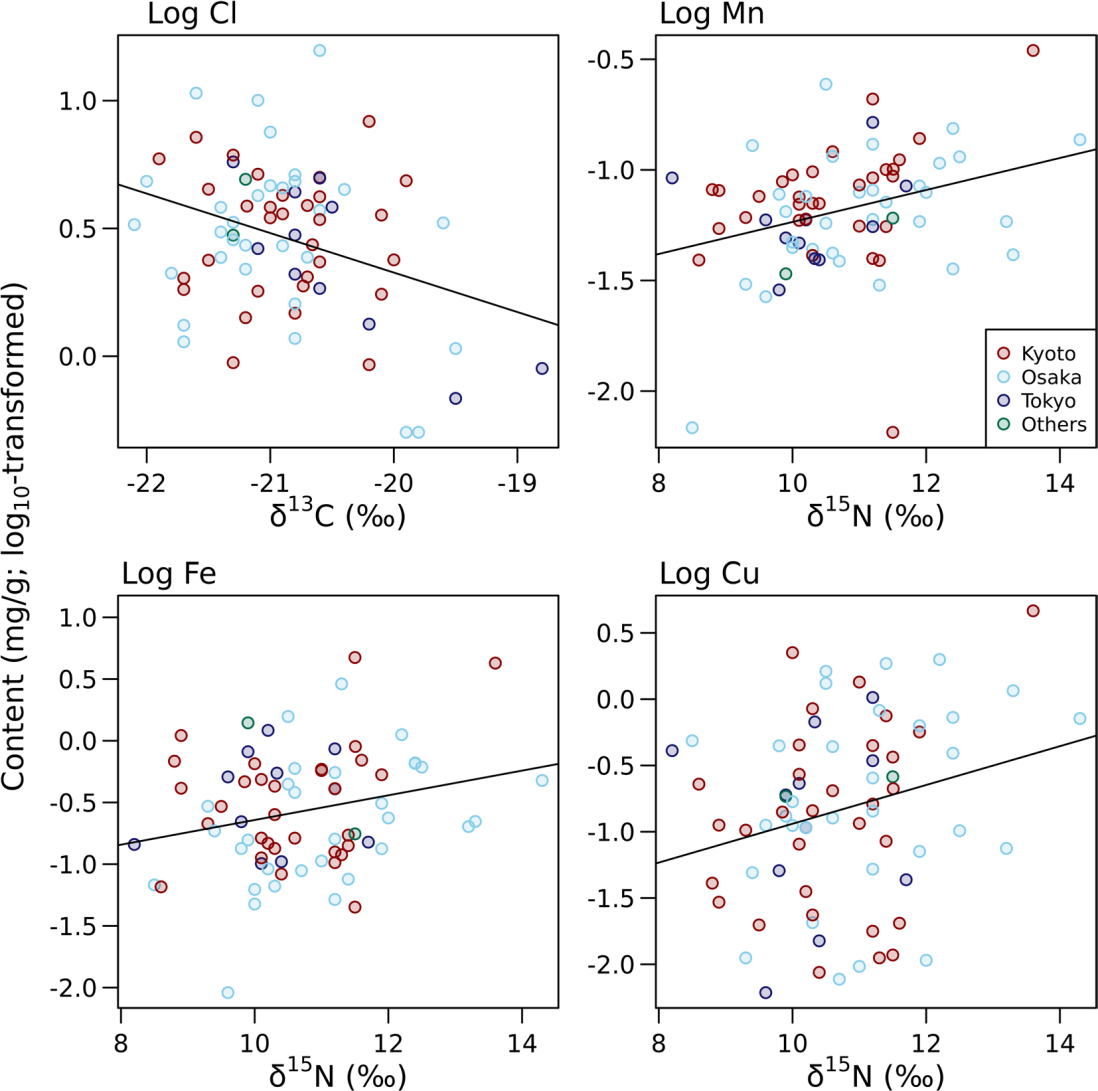
Relationships between stable isotope ratios (*δ*^13^C and *δ*^15^N) and the selected elemental contents in hair embedded in book paper. Elemental contents were measured by PIXE analysis and log_10_-transformed. Regression lines are drawn for pooled data across locations. Colors indicate the different locations (Kyoto, Osaka, Tokyo, and five other provinces). Only relationships showing significant correlations are presented; the complete set of isotope–element relationships is provided in Fig. S2.

Relationships between stable isotope ratios and elemental concentrations, assessed by linear regression using *δ*^13^C or *δ*^15^N as predictors of the 75 historical dataset pooled across all locations, showed selective but informative associations (Fig. 4; Fig. S2). *δ*^13^C correlated negatively with Cl (*t* = − 2.98, *p* = 0.004). *δ*^15^N correlated positively with Mn (*t* = 2.81, *p* = 0.006), Fe (*t* = 2.16, *p* = 0.034), and Cu (*t* = 2.22, *p* = 0.029). No other isotope–element combinations showed significant associations (*p* > 0.05).

## Discussion

### Mineral-rich diets in early modern to modern urban Japan

Elemental analyses of human hair preserved in historical book covers reveal that urban populations in early modern to modern Japan were exposed to substantially higher levels of multiple elements than those observed today (Fig. 2). Compared with contemporary reference samples, historical hair showed significantly elevated concentrations of Cl, K, Ca, Mn, Fe, Cu, and Pb, while S and Zn were higher in contemporary hair and Si showed no detectable difference. These differences are large and consistent across locations, with only modest interaction effects between era and location.

This pattern is notable because the isotopic evidence indicates that diets during this period were relatively simple, being dominated by C_3_ staples (e.g., rice and wheat), with marine fish as the principal nitrogen source (Fig. 3a–b)^15^. This interpretation is consistent with historical studies of Japanese food culture, which have emphasized the cultural centrality of rice, particularly in urban settings, together with the supplementary role of inexpensive marine fish such as sardine and mackerel in the diets of urban commoners^21,22^. Despite this apparent dietary narrowness, and despite living in large urban centers long after the transition to agriculture, concentrations of multiple elements in hair were higher than in contemporary populations. The results therefore suggest that mineral availability in early modern to modern diets was not determined by dietary diversity but was shaped by other factors associated with food production, processing, preservation, and preparation. Identifying the mechanisms behind this elevated mineral availability is therefore essential for interpreting long-term dietary change.

### More straightforward explanations for selected elements

The elemental concentrations in hair reflect multiple influences associated with food processing, preservation, cooking, and everyday material practices, rather than a single dietary factor. For a small subset of elements, however, the dominant causes can be interpreted more straightforwardly, particularly when they are linked to preservation or non-dietary exposure.

**Fig. 5:**
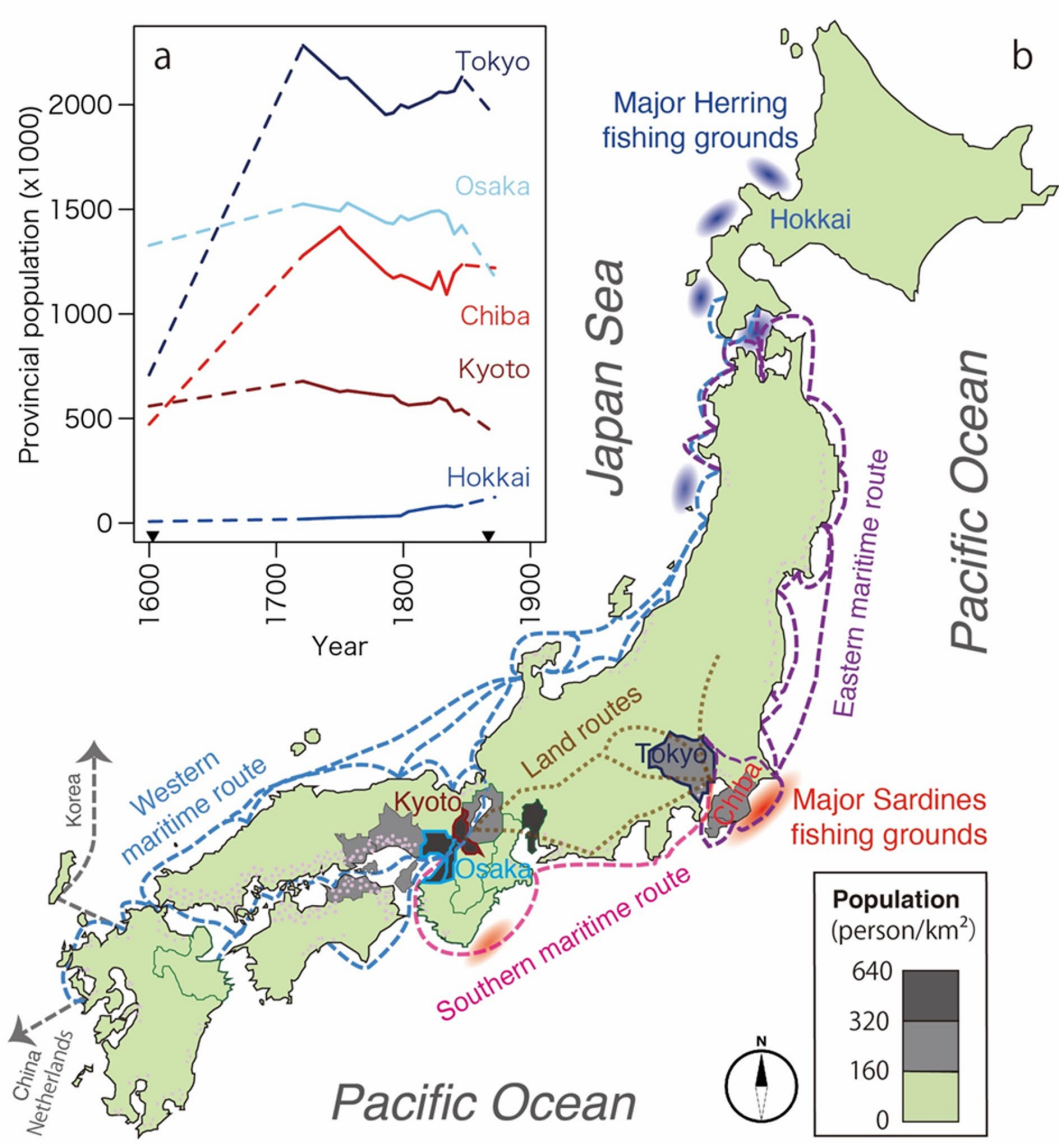
Historical background of early modern Japan. a: Temporal changes in population sizes of the provinces corresponding to Kyoto (Yamashiro), Osaka (Settsu, Izumi, Kawachi), and Tokyo (Musashi), and those associated with two major fishing grounds, namely Chiba (Awa, Kazusa, Shimousa) and Hokkai (Ezo), based on population estimates adjusted for regime differences in statistical records^2^. Dashed lines connect estimates based on statistical data from different regimes, with triangles along the X-axis marking regime transitions. b: Map of population density for 1786. Dashed lines of different colors on the map represent maritime distribution routes developed in the three regions. Dotted lines represent five land routes. Red and blue circles indicate major fishing grounds for sardines and herring, respectively. The five areas enclosed by green lines are provinces—other than Kyoto, Osaka, and Tokyo—where book samples used in this study, with hair embedded in the paper, were printed. Pale pink dotted bands mark coastal areas historically renowned for salt production, most prominently along the Seto Inland Sea, which accounted for approximately 80% of national output by 1879^3^. The map was created by the authors in Adobe Illustrator 2023 (version 27.6.1; Adobe, San Jose, CA, USA; https://www.adobe.com/jp/products/illustrator.html).

Salt preservation provides an easy explanation for the exceptionally high Cl concentrations in historical hair. Before refrigeration, NaCl functioned not only as a seasoning but also as an essential preservative, and historical diets would therefore have been exposed to substantially higher Cl inputs than contemporary diets shaped by salt-reduction efforts. Regional contrasts observed among historical samples are also consistent with this interpretation. Kyoto and the surrounding cultural sphere, including Osaka, developed culinary traditions characterized by salt-fermented fish products such as narezushi, as well as the use of relatively salty usukuchi soy sauce, whereas northern regions relied more heavily on dried fish and milder koikuchi soy sauce^22,28–30^. Tokyo, moreover, depended heavily on salt transported from the Seto Inland Sea over long distances (Fig. 5), at prices substantially higher than in coastal production regions^31,32^, which may also have influenced local patterns of use.

A second element that can be interpreted more straightforwardly is Pb, which likely reflects non-dietary exposure associated with everyday urban life. Its markedly higher concentrations in historical hair are most plausibly explained by exposure to white face powder (oshiroi), a common cosmetic that contained substantial amounts of lead, consistent with osteoarchaeological evidence of lead contamination in early modern populations^33^, although other exogenous urban sources of lead cannot be excluded. These patterns suggest that elevated elemental concentrations observed in early modern urban Japan were shaped not simply by what people ate, but by how food was processed, preserved, and prepared, as well as by material practices surrounding daily life.

### Isotopic context for dietary mechanisms

The remaining elemental patterns can be interpreted only in the context of the dietary framework revealed by isotope evidence from early modern to modern Japan. Historical hair consistently shows lower *δ*^13^C and higher *δ*^15^N values than contemporary Japanese hair and other contemporary reference populations (Fig. 3)^15^, indicating diets dominated by C_3_ staples such as rice and wheat, together with marine fish as the principal nitrogen source^16–20,26,27^. This pattern accords with established knowledge of Japanese dietary history, including the long-standing cultural centrality of rice and the limited role of mammalian meat^21,22^. This interpretation suggests that the likely explanations for the elevated elemental concentrations observed in historical hair should be sought first in these two major dietary components identified by isotope evidence: C_3_ staples and marine fish.

The clearest signal concerns rice polishing. Si and K, which are concentrated in the outer layers of rice grains and are readily removed during polishing (Table S1)^34–36^, showed higher concentrations in historical hair than in contemporary hair and declined through time across the historical dataset, consistent with the spread and intensification of rice polishing. The higher K concentrations observed in the five other provinces further suggest that rice polishing advanced earlier or more intensively in the major cities than in provincial towns, possibly owing to earlier access to urban infrastructure and milling technology.

At the same time, not all elements in the historical dataset followed the pattern expected from polishing alone. Ca, Mn, Fe, Cu, and Zn are also known to decrease when rice is polished (Table S1)^34–36^, and all of these elements except Zn were higher in historical hair than in contemporary hair. Yet these elements did not show comparable long-term declines in the historical dataset. This discrepancy suggests that dietary mineral availability in early modern to modern urban diets was sustained by additional inputs that offset losses from grain polishing. One plausible contributor is the use of metal cookware, which may have added Fe and Cu during food preparation^37^. The location-specific temporal trajectories observed for Fe and Cu in the historical dataset likewise imply that changes in food preparation and household material culture did not occur uniformly across regions, but followed distinct local pathways shaped by urban access and culinary practice. These explanations, however, cannot account for the relatively low Zn concentrations or the persistence of Ca and Mn in historical hair. These remaining patterns require further interpretation in light of the isotopic evidence considered below.

### Isotopic evidence refining elemental interpretations

Temporal trends in isotope ratios of the historical hair provide further clues to the factors shaping elemental concentrations. They indicate that this dietary system changed gradually rather than abruptly (Fig. 3). The decline in *δ*^13^C suggests diminishing contributions of C_4_ crops such as millet and foxtail millet, consistent with the consolidation of rice-based C_3_ staples. At the same time, the increase in *δ*^15^N indicates growing reliance on ^15^N-enriched nitrogen sources, most likely marine fish. Although marine foods generally have higher *δ*^13^C values than terrestrial C_3_ staples, the temporal decline in *δ*^13^C can still be reconciled with increasing marine inputs if the reduction in C_4_ plant consumption was the stronger effect. Likewise, the increase in *δ*^15^N is unlikely to reflect shifts in the balance between C_3_ and C_4_ staples alone, because these crops occupy similar trophic positions, whereas marine fish provide substantially higher *δ*^15^N values. Freshwater fish are not emphasized here because their isotope values vary widely with local environmental conditions, and they were likely less important than marine fish in the diets of urban commoners. This interpretation of increasing marine fish contribution parallels the documented expansion of highly productive coastal fisheries, especially the 18th -century sardine fisheries in Chiba and the 19th -century herring fisheries in Hokkaido, enabled by improvements in transport and distribution networks (Fig. 5)^38,39^. These fisheries were sufficiently productive that fishing regions experienced substantial population concentration during periods of abundant catches (Fig. 5a)^40^. The modest regional offsets in *δ*^13^C, with Osaka earlier and Tokyo later, are also consistent with historical differences in staple crop composition^41^ and rice-centered distribution systems associated with the land tax system, through which rice collected from rural domains circulated via Osaka as the principal commercial hub linking major urban centers. Taken together, these trends provide an important context for interpreting the element-specific patterns discussed below.

More directly, the isotope–element relationships help refine the elemental interpretations (Fig. 4). The positive associations of *δ*^15^N with Mn, Fe, and Cu are particularly informative. These are precisely the elements that did not decline in the way expected from rice polishing alone, suggesting that increasing dependence on marine fish may have compensated for mineral losses associated with refined staples. Marine fish are relatively rich in Mn, Fe, and Cu compared with rice-based staples (Table S1), making them a plausible nutritional source of these elements. Importantly, both elevated *δ*^15^N and the persistence of Mn, Fe, and Cu in human hair may reflect not only direct consumption of marine fish but also the widespread historical practice of applying fish-derived fertilizers to paddy fields^38,39^; these mechanisms are not mutually exclusive. In the present study, these correlations therefore strengthen the interpretation that the mineral richness of early modern urban diets emerged not despite the dominance of rice and fish, but through the mineral contributions of marine resources within a rice-based dietary system.

By contrast, the negative association between *δ*^13^C and Cl is best understood cautiously, because both variables also vary geographically. It likely reflects a regional correspondence between stronger reliance on C_3_ staples and higher salt use rather than an individual-level dietary mechanism.

A different interpretation may apply to Zn, the only trace element that was higher in contemporary hair than in historical samples. Unlike Mn, Fe, and Cu, Zn does not fit the pattern expected from rice polishing or increased fish contribution. Instead, its elevation in contemporary samples may reflect the greater contribution of mammalian meat to contemporary diets. Previous isotopic studies have shown that contemporary Japanese hair exhibits *δ*^13^C values shifted toward more Americanized dietary patterns, including increased consumption of beef and pork (Fig. 3)^16,17^, foods that had been largely excluded from Japanese diets by both cultural taboo and formal prohibition until the end of the early modern period^21^. This interpretation is consistent with food composition data, because beef and pork contain substantially more Zn than rice-based staples and fish products (Table S1). This explanation suggests that the higher Zn concentrations in contemporary hair may reflect the dietary incorporation of mammalian meat.

### Implications for contemporary micronutrient availability

Globally, mineral deficiencies remain a major public health concern, affecting over two billion people^1,2^. In Japan, national nutrition surveys^3^ indicate that actual intakes of Ca, Fe, Zn, and Cu remain below the recommended levels set out in national dietary reference standards^4^. Such widespread insufficiency has sometimes been interpreted as a mismatch between contemporary diets and human physiology, evolved under the diverse and mineral-rich diets of hunter–gatherers from approximately 150,000 to 10,000 years ago^5^.

Against this broader context, the elevated elemental concentrations observed in our historical hair are noteworthy. They suggest that relatively simple diets in the major cities of early modern to modern Japan supported greater mineral availability, even long after the transition to agriculture but before industrialization transformed food supply, distribution, and preservation systems. As discussed above, this pattern likely reflects a combination of factors related to food processing, preservation, cooking practices, and material culture rather than simple dietary diversity. Historical dietary systems based primarily on rice and marine fish (Fig. 3) appear capable of sustaining substantial mineral availability under certain technological and culinary conditions.

Hair elemental content represents the surplus portion retained after essential absorption and excretion and is therefore not linearly related to total dietary intake. In addition, both deficiency and excess intake can pose health risks^5,6^. This caution is particularly relevant for elevated Cl, likely reflecting salt preservation, and elevated Pb, likely reflecting exposure to white face powder, rather than nutritional advantage. The present study therefore does not imply that early modern to modern diets were inherently “healthier”, nor does it attempt to reconstruct their exact nutritional composition. Nevertheless, the contrast between historical and contemporary mineral signals remains striking. Rather than prescribing specific dietary practices, these findings highlight how food processing, preservation, and preparation practices can substantially influence mineral availability, offering historical perspective for current discussions of nutritional adequacy.

### Books preserving bioarchives: the promise of libraromics

Beyond nutritional implications, this study also illustrates the broader potential of libraromics, the analysis of biological materials preserved within books and other cultural artifacts. Hair embedded in historical book paper preserves biochemical information that is rarely accessible through conventional archaeological materials such as bones or teeth. Compared with such sources, book-derived hair offers several advantages, including minimally destructive sampling procedure described in Materials and methods, stable preservation of biochemical signals in keratin, and the possibility of assembling large datasets from well-dated cultural objects.

These characteristics highlight the scientific value of books preserving biological archives. In an era of large-scale digitization, when the informational value of texts is increasingly separated from the physical objects that contain them, the biological traces preserved in historical materials risk being overlooked. Yet the present results demonstrate that such artifacts can retain biochemical signals reflecting past diets, environments, and human practices. In this sense, the embedded hair functions as a bioarchive, while the book itself serves as a time capsule that preserves this information without the ethical and cultural concerns often associated with excavating human remains.

Several limitations nevertheless warrant consideration. The present dataset is biased toward the three major cities, reflecting the survival and circulation of urban publications, although the inclusion of five provincial towns demonstrates that the approach can extend beyond metropolitan contexts. Individual attributes such as sex and age cannot be determined from the available samples, making large sample sizes essential for population-level inference. Because the recovered hairs lack roots, DNA recovered from them is unlikely to support reliable individual-level identification, including sex determination. PIXE analysis also restricts the set of quantifiable elements due to overlapping X-ray peaks. The specific elements excluded depend on the analytical configuration; in the present study, Na, Mg, and Sr were not quantified. These elements could potentially reveal variation in water sources; this limitation is relatively minor in Japan because most historical books originated from lowland cities characterized by uniformly soft (low-mineral) water. The isotope–element relationships observed here suggest that marine resources played a compensatory role in maintaining mineral availability, although the relative importance of direct fish consumption versus the use of fish-derived fertilizers remains unresolved. Future analyses of historical rice samples may help clarify this distinction. More broadly, expanding libraromic approaches to other archived biological materials may open new opportunities to reconstruct interactions among diet, environment, and cultural practice across historical timescales.

## Materials and methods

### Historical books and embedded hair samples

This study analyzed human hair embedded in the recycled paperboard used in the covers of traditional Japanese bound books (wahon) produced in Japan during the early modern to modern period (17th to 19th centuries)^42–45^. Because many books preserve colophon information indicating the year and place of publication, the embedded hair can be linked to specific cities and time periods (Fig. 1).

In Japan, commercial publishing expanded from the 17th century onward under conditions of long-term political stability and the growth of urban literacy and leisure culture, producing a large volume of printed materials in the three major metropolitan centers of Kyoto, Osaka, and Edo (Tokyo)^42,45,46^. These cities also formed the principal centers of recycled paperboard production and circulation. From a bibliographical perspective, the embedded hair is thought to have been incorporated incidentally when discarded paper bearing loose hairs was repulped, or to have been mixed intentionally into pulp to reinforce recycled sheets. In both cases, the embedded hair is reasonably interpreted as having originated from people living in the same cities and periods in which the books were produced. This interpretation is supported by the fact that transportation and long-term storage were costly for such inexpensive cover materials, so recycled paper was mostly produced and reused within the same urban commercial systems^47^. Furthermore, because commercial publishing, paper recycling, and book circulation in this period were concentrated in urban settings, the resulting dataset is interpreted principally as a record of dietary and environmental exposure among urban commoners rather than rural populations.

Potential contamination from materials used in papermaking, paper recycling, or cover production is unlikely to have been a major influence on the elemental compositions of the embedded hairs. In traditional papermaking, the materials used were primarily polysaccharide-based, and hard water was generally avoided because it interfered with sheet formation. In paper recycling, even the use of such polysaccharide-based materials is considered to have been limited. In addition, adhesive in traditional Japanese bookbinding was typically applied only in limited amounts at the outer edges of the cover materials rather than throughout the recycled paperboard itself.

Books examined in this study were selected on a book-set basis, following our pilot study^15^. A book set is the unit in which volumes of the same title were originally printed, sold, and resold together, and in Japanese woodblock books this unit is more appropriate than individual volumes because volumes within a set derive from the same printing event and share the same recycled cover board context. Among a total of approximately 70,000 volumes screened, 810 book sets were examined in detail, of which 765 sets were from the holdings of the Ryukoku University Omiya Library (Fig. 1a), 23 sets were retained from our pilot study^15^, 20 sets were newly purchased from secondhand bookstores, and 2 sets were provided as hair-only samples from the Tajimi City Library Local History Room. About half of the surveyed book sets contained extractable hair (403 book sets). When hair was present, small portions were removed from the recycled board using minimally invasive procedures, typically by gently pulling individual hairs out with forceps. This was generally possible without adding moisture, without unintentionally removing surrounding paper fibers, and without breaking the hairs. The recovered specimens were identified as human head hair using, when necessary, microscopic criteria for human hair identification^48^, including medulla-to-shaft ratio, straight morphology, and unsplit tips.

For each book set, printing year and printing location were determined through bibliographic validation of the colophon^15^, and only sets with a clearly identified printing city and a discrepancy of less than 10 years between the stated publication year and the estimated printing year were retained for subsequent analyses (*n* = 233). The reliability of this bibliographic validation was demonstrated in our pilot study, in which independent examinations by two investigators indicated that the estimated printing years differed by less than 30 years^15^. For analyses of temporal trends in elemental concentrations and stable isotope ratios, the bibliographically corrected printing year was used as the temporal variable. Most validated sets originated from Kyoto, Osaka, and Edo (Tokyo), mirroring the historical concentration of publishing in the three metropolises. Detailed information on the screening outcomes of sampled book sets and recovered hair samples is provided in Fig. S3–S4.

### Contemporary reference hair samples

For comparison, contemporary hair samples were collected specifically for PIXE analysis, as stable isotope data for contemporary Japanese populations are already available from a previous study^17^. Sampling targeted residents of Kyoto, Osaka, and Tokyo across a broad range of ages and both sexes to represent urban population diversity. Potential donors completed a short questionnaire recording age, gender, dietary habits (including whether they were vegetarian or vegan), and birthplace of themselves and their parents. Individuals were excluded if they or their parents were born outside Japan or if they reported vegetarian or vegan diets. Hair was provided anonymously, with each collaborator collecting multiple samples from acquaintances. All methods involving contemporary human participants were carried out in accordance with relevant guidelines and regulations of Ryukoku University and were approved in advance by the Research Ethics Committee for Studies Involving Human Subjects at Ryukoku University (Approval No. 2019-07). Informed consent was obtained from all participants prior to sample collection. Sampling was conducted between January and December 2020, and the collected hair likely reflects dietary practices over the preceding years depending on hair length.

Following collection, 40 samples were selected to maintain diversity within each city, covering individuals aged 18 years to their 60s: 15 in Kyoto (8 females, 7 males), 13 in Osaka (7 females, 6 males), and 12 in Tokyo (7 females, 5 males), with representation across sex and age classes in each city. Thus, the contemporary individuals used as references in this study were urban adult residents with balanced representation across sex and age classes and did not include vegetarians or vegans.

### Elemental analysis (PIXE)

Elemental concentrations in hair samples were determined using a micro-PIXE analytical system at the electrostatic accelerator facility of the National Institute of Radiological Sciences, Japan^49^. PIXE provides low-background X-ray spectra and enables simultaneous, essentially non-destructive multi-element analysis of solid biological samples without chemical digestion, making it well suited for individual hairs.

Of the 233 bibliographically validated book sets, 104 containing hair strands longer than 2 cm were selected for PIXE analysis based on analytical requirements. PIXE measurements were performed on a single hair from each selected book set, because the analytical setup required one strand long enough to span the opening of the carbon-based sample holder. Hair fragments recovered from the recycled paperboards were first gently cleaned with ethanol to remove loosely attached surface particles. Individual hairs were then mounted on clean carbon-based sample holders and irradiated with a focused proton beam. Characteristic X-rays emitted from the samples were detected and used to quantify elemental concentrations. Elements reliably quantified in this study included Si, S, Cl, K, Ca, Mn, Fe, Cu, Zn, and Pb. Quantification was performed using an external standard method based on reference hair samples previously analyzed by PIXE^50,51^. Elemental concentrations were calculated from the detected X-ray spectra using standard PIXE analytical protocols. Because the method allows direct analysis of untreated samples, the procedure minimized the risk of elemental loss or contamination associated with wet-chemical preparation.

Each sample was measured once because of the fragility of individual hair strands. Elements with poorly resolved peaks or inconsistent quantification (e.g., Na, Mg, Br, and Hg) were excluded from further analyses. Although Sr was successfully quantified, most measurements were below the limit of quantification, particularly in contemporary hair samples; therefore, Sr was excluded from subsequent analyses. Data for Cl and Mn were unavailable for two samples each because of analytical constraints. Additional technical details of the PIXE settings are provided in Table S2.

### Stable isotope analysis

Stable carbon and nitrogen isotope ratios of hair samples were measured using a Delta V Advantage isotope ratio mass spectrometer (Thermo Fisher Scientific, Bremen, Germany) coupled to a Flash EA 1112 elemental analyzer via a Conflo IV interface at the Faculty of Advanced Science and Technology, Ryukoku University. Isotopic analyses were performed using standard EA–IRMS procedures for keratinous materials^10,11,15^.

Of the 233 bibliographically validated book sets, 161 with a total recovered hair length of at least 8 cm were selected for isotope analysis based on analytical requirements. Because our pilot study showed that within-book-set isotopic variation was much smaller than between-book-set variation^15^, isotope measurements were based on one or more hairs pooled within each book set when necessary to obtain sufficient material. Both isotope and PIXE data were available for 75 sets, with PIXE performed first because it is largely non-destructive. Hair fragments recovered from the recycled paperboards were cleaned using a chloroform–methanol solution (2:1) followed by ultrasonic treatment for 10 min to remove external contaminants, and were subsequently dried at 60 °C for at least 12 h prior to analysis. Cleaned hair samples were cut into small segments and weighed (approximately 0.55–0.65 mg) into tin capsules for isotopic measurement. This mass typically corresponded to hair lengths of approximately 8–12 cm. Because human scalp hair grows at approximately 1 cm per month^52^, isotope ratios represent dietary inputs integrated over the months preceding hair formation.

Carbon and nitrogen isotope compositions are reported in *δ* notation relative to international standards (Vienna Pee Dee Belemnite for carbon and atmospheric N_2_ for nitrogen). Analytical precision, monitored using laboratory reference materials (alanine: *δ*^13^C = − 19.6 ± 0.2‰ and *δ*^15^*N* = 1.6 ± 0.2‰, histidine: *δ*^13^C = − 10.7 ± 0.2‰ and *δ*^15^*N* = − 7.6 ± 0.2‰), was better than ± 0.2‰ for both *δ*^13^C and *δ*^15^N. All analyzed samples yielded atomic C/N ratios of 3.09 ± 0.08 (mean ± SD), consistent with the expected range for keratin. To correct for the Suess effect (the decline in atmospheric *δ*^13^C resulting from fossil fuel emissions), *δ*^13^C values of contemporary reference datasets were adjusted by adding 1.6‰ for the 1980s dataset and 2.0‰ for the 2000s dataset, aligning them with the atmospheric baseline of the early modern to modern period (17th to 19th centuries).

### Statistical analyses

Statistical significance was evaluated using a threshold of *p* < 0.05. All statistical analyses were conducted in R version 4.4.2^53^.

Elemental concentrations were compared between historical and contemporary samples using linear models that included era, location, and their interaction as explanatory variables. Because the distributions of most elemental concentrations were approximately log-normal, all elements except S were log_10_-transformed prior to analysis (Fig. S5). Additive models were used when the interaction term did not improve model fit.

Temporal trends in elemental concentrations and stable isotope ratios were examined only for historical hair samples using linear models with corrected printing year, location, and their interaction as explanatory variables. Additive models were used when interaction terms did not improve model fit.

Relationships between isotope ratios and elemental concentrations were examined using Pearson correlation analyses to explore potential dietary mechanisms underlying the observed elemental patterns. These analyses focused on associations between *δ*^13^C or *δ*^15^N and individual elemental concentrations measured in the same historical hair samples.

Exploratory analyses of dataset structure and additional bibliographic characteristics of sampled books were conducted during data preparation but are not central to the present study; these analyses, together with extended statistical results, are provided in Fig. S3–S4.

## Supplementary Information

Below is the link to the electronic supplementary material.

Supplementary Material 1

Supplementary Material 2

## Data Availability

The original data sheets are provided as Supplementary Information. Additional data are available from the corresponding author upon reasonable request.
