# Supplementary material for: Book-embedded hair reveals mineral-rich diets among urban commoners in early modern to modern Japan
Source: Sci Rep. 2026 Aug 17;16:23469. doi: 10.1038/s41598-026-63908-y (PMC13483190; doi:10.1038/s41598-026-63908-y)
Supplement: Supplementary file 1 [file 41598_2026_63908_MOESM1_ESM.docx]

**Supplementary Information for**

Book-embedded hair reveals mineral-rich diets among urban commoners in early modern to modern Japan

Atsushi Maruyama^1*^, Shota Kuwaki^1^, Shuntaro Kimura^1^, Yuki Sanada^1^, Masakazu Oikawa^2^, Shoji Futatsukawa^3^, Yukihiro Kohmatsu^4^, Atsushi Iriguchi^5^

1.	Faculty of Advanced Science and Technology, Ryukoku University, Seta-oe, Otsu, Shiga, 520-2194, Japan.

2.	National Institute for Quantum Science and Technology, Institute for Quantum Medical Science, 4-9-1 Anagawa, Inage-ku, Chiba 263-8555, Japan

3.	Alpha Tau Medical K.K., 1-9-15 Nihonbashimuromachi, Chiyoda-ku, Tokyo 103-0022, Japan.

4.	The Ministry of the Environment, Kyushu Regional Environment Office, 4F, Kumamoto Regional Joint Government Building B, Kasuga 2-10-1, Nishi Ward, Kumamoto City, Kumamoto Prefecture 860-0047, Japan.

5.	National Institute of Japanese Literature, Midori-cho, Tachikawa, Tokyo, 190-0014, Japan.

*Corresponding author: maruyama@rins.ryukoku.ac.jp

**This document includes:**

Table S1. Mineral contents

Table S2. PIXE details

Fig. S1. Element MDS

Fig. S2. Isotope–element plots

Fig. S3. Book-field histogram

Fig. S4. Hair details

Fig. S5. Element histograms

Supplementary Information References

Supplementary Table S1. Mineral contents (mg/100 g dry weight) in brown, semi-polished, and white rice, as well as selected fish and meat items. Values are based on the MEXT Standard Tables of Food Composition in Japan, 8th Edition [1], and converted from fresh weight to dry weight for comparability across food categories.

|  | **K** | **Ca** | **Mn** | **Fe** | **Cu** | **Zn** | **NaCl equivalent** | **Japanese term in the DB** |
| --- | --- | --- | --- | --- | --- | --- | --- | --- |
| **Rice** |  |  |  |  |  |  |  |  |
| **Brown rice** | 270 | 11 | 2.42 | 2.5 | 0.32 | 2.1 | 0 | 穀類/こめ/［水稲穀粒］/玄米 |
| **Semi-polished rice** | 176 | 8 | 1.65 | 1.8 | 0.28 | 1.9 | 0 | 穀類/こめ/［水稲穀粒］/半つき米 |
| **White rice** | 105 | 6 | 0.95 | 0.9 | 0.26 | 1.6 | 0 | 穀類/こめ/［水稲穀粒］/精白米/うるち米 |
| **Fish** |  |  |  |  |  |  |  |  |
| **Dried sardine** | 1,035 | 969 | 0.22 | 9.7 | 0.46 | 4.0 | 8,370 | 魚介類/<魚類>/（いわし類）/まいわし/丸干し |
| **Dried herring** | 1,091 | 168 | 0.10 | 3.8 | 0.25 | 3.3 | 1,015 | 魚介類/＜魚類＞/にしん/身欠きにしん |
| **Meat** |  |  |  |  |  |  |  |  |
| **Beef** | 674 | 16 | NA | 6.2 | 0.16 | 13.5 | 518 | 肉類/＜畜肉類＞/うし/［ひき肉］/生 |
| **Pork** | 824 | 17 | 0.03 | 2.8 | 0.20 | 8.0 | 284 | 肉類/＜畜肉類＞/ぶた/［ひき肉］/生 |

Si, S, Cl, and Pb are not included in the MEXT food composition database.

NaCl equivalent was calculated from sodium content using a conversion factor of 2.54 (NaCl/Na).

Supplementary Table S2. Additional technical details of the PIXE instrumentation and analytical procedures.

| **Category** | **Description** |
| --- | --- |
| Sample mounting | Hair strands were mounted in metal-free carbon-based holders and stretched across the aperture without adhesives |
| Accelerator | 1.7 MV Tandetron accelerator (Model 4117MC+, High Voltage Engineering Europa B.V.) |
| Ion microbeam system | OM-2000 ion microbeam endstage |
| Data acquisition | OM-DAQ2007 system (Oxford Microbeams Ltd.) |
| Detectors | Si(Li) detector (Sirius80, Gresham) and CdTe diode detector (XR-100T-CdTe, Amptek) |
| Measurement environment | Vacuum chamber maintained at approximately 10^–5^ Pa |
| Proton beam | 3.0 MeV proton beam with a beam current of approximately 100 pA |
| Irradiation conditions | Approximately 30 min irradiation (~200 nC total charge) |
| Beam spot size | Approximately 1 µm |
| Spectral processing | Spectra processed using OM-DAQ 2007 with corrections for background, peak overlap, and detector efficiency |

Fig. S1. Multidimensional scaling (MDS) plots based on Aitchison distances among hair samples, calculated from centered log-ratio-transformed contents of ten elements measured by PIXE analysis. a: MDS scatter plot, with point size representing the bibliographically corrected year of woodblock printing and arrows indicating the contribution of each element to the MDS axes. b, c: Temporal changes in MDS 1 (b) and MDS 2 (c), plotted against the bibliographically corrected printing year. Colors represent different locations of book printing. Permutational multivariate analysis of variance (PERMANOVA) detected no significant interaction between year and location. Year had a significant effect on overall elemental composition (*F*_1,99_ = 7.38, *p* = 0.001), whereas location showed a weaker effect (*F*_3,99_ = 1.49, *p* = 0.082). Pairwise comparisons indicated a significant difference between Tokyo and Kyoto (*F*_1,99_ = 2.16, p = 0.040), whereas other location pairs were not significant (*p* > 0.2). These results indicate a marked temporal shift and only modest spatial divergence in elemental composition.

Fig. S2. Relationships between stable isotope ratios (*δ*^13^C and *δ*^15^N) and the elemental contents in hair embedded in book paper. Element contents were measured by PIXE analysis and log_10_-transformed, except for sulfur (S). Colors indicate the different locations (Kyoto, Osaka, Tokyo, and five other provinces). Regression lines are drawn for pooled data across locations, with solid lines indicating significant relationships and dotted lines indicating non-significant ones. Linear regressions detected a significant negative association between *δ*^13^C and Cl (*t* = –2.98, *p* = 0.0039), and significant positive associations between *δ*^15^N and Mn (*t* = 2.81, *p* = 0.0063), Fe (*t* = 2.16, *p* = 0.034), and Cu (*t* = 2.22, *p* = 0.029). No other isotope–element combinations showed significant relationships (*p* > 0.05).

Fig. S3. Histograms of book sets in ten subject categories (e.g., story, religions, history, etc.) among bibliographically validated samples from Ryukoku University Omiya Library whose paper contained embedded hair used for stable isotope and/or PIXE analyses, plotted against the bibliographically corrected year of woodblock printing for Kyoto, Osaka, Tokyo, and five other provinces. Only book sets whose estimated printing year differed by less than 10 years from the stated publication year are included. Literary works were the most frequent among the analyzed sets, whereas Buddhist and historical books, although abundant in the Omiya Library collection, rarely contained extractable hair. The number of analyzed book sets increased significantly through time (GLM slope: *z* = 3.56, *p* < 0.001) and appeared earliest in Kyoto, followed by Osaka (*z* = –1.58, *p* = 0.113), Tokyo (*z* = –4.53, *p* < 0.001), and the other provinces (*z* = –5.08, *p* < 0.001). Because all samples were derived from Ryukoku University Omiya Library, part of a Kyoto-based Jodo Shinshu Buddhist university, some bias in subject composition and temporal representation is possible.

Fig. S4. Details of embedded hair analyzed in this study. Panels (a–d) share the same axes: total hair length per book set (a, b), number of hairs per book set (c, d), bibliographically corrected year of woodblock printing (a, c), and printing location (b, d). Only book sets whose estimated printing year differed by less than 10 years from the stated publication year are included. Each book set yielded 1–100 strands, totaling 1–120 cm of hair. Total hair length differed significantly among locations, with Tokyo < Kyoto < Osaka (Tukey HSD: p < 0.05), whereas the number of hairs showed no significant spatial or temporal variation (p > 0.05). Because most samples were derived from Ryukoku University Omiya Library, located in Kyoto since 1639, some bias in temporal and spatial representation is possible.

**Fig. S5. Histograms of the content of 11 elements in hair embedded in book paper (a) and hair of contemporary people (b).** For each sample type, the upper and lower panels show the original and log₁₀-transformed content (mg/g), respectively. Element contents were measured by particle-induced X-ray emission (PIXE) analysis. For samples with undetected element content, the minimum value of each element was assigned before log₁₀ transformation. Sulfur (S), the most important structural component of keratin in hair among the 11 measured elements, did not follow a log-normal distribution and therefore was not transformed. Strontium (Sr) was excluded from further analyses because, despite successful quantification, it yielded an excess of zero values—even in book-derived hairs containing relatively high concentrations of other elements—and nearly all contemporary hairs showed zero values.

**Supplementary Information References**

1.	Ministry of Education, Culture, Sports, Science and Technology (MEXT), Standard Tables of Food Composition in Japan (8th Revised Edition, Supplement 2023). (MEXT, Tokyo, Japan, 2023), (In Japanese). https://www.mext.go.jp/a_menu/syokuhinseibun/mext_00001.html. Accessed 23 March 2026.
